# Reducing decision errors in the paired comparison of the diagnostic accuracy of screening tests with Gaussian outcomes

**DOI:** 10.1186/1471-2288-14-37

**Published:** 2014-03-05

**Authors:** Brandy M Ringham, Todd A Alonzo, John T Brinton, Sarah M Kreidler, Aarti Munjal, Keith E Muller, Deborah H Glueck

**Affiliations:** 1 Center for Cancer Prevention and Control Research, University of California, Los Angeles, 650 Charles Young Drive South, Room A2-125 CHS, Los Angeles CA 90095, USA; 2 Department of Preventive Medicine, University of Southern California, 440 E. Huntington Dr, 4th floor, Arcadia CA 91006, USA; 3 Department of Biostatistics and Informatics, Colorado School of Public Health, University of Colorado Anschutz Medical Campus, 13001 E. 17th Place, Aurora CO 80045, USA; 4 Department of Health Outcomes and Policy, 1329 SW 16th St., Gainesville FL 32608, USA

**Keywords:** Cancer screening, Differential verification bias, Area under the curve, Type I error, Power, Paired screening trial, Receiver operating characteristic analysis

## Abstract

**Background:**

Scientists often use a paired comparison of the areas under the receiver operating characteristic curves to decide which continuous cancer screening test has the best diagnostic accuracy. In the paired design, all participants are screened with both tests. Participants with suspicious results or signs and symptoms of disease receive the reference standard test. The remaining participants are classified as non-cases, even though some may have occult disease. The standard analysis includes all study participants, which can create bias in the estimates of diagnostic accuracy since not all participants receive disease status verification. We propose a weighted maximum likelihood bias correction method to reduce decision errors.

**Methods:**

Using Monte Carlo simulations, we assessed the method’s ability to reduce decision errors across a range of disease prevalences, correlations between screening test scores, rates of interval cases and proportions of participants who received the reference standard test.

**Results:**

The performance of the method depends on characteristics of the screening tests and the disease and on the percentage of participants who receive the reference standard test. In studies with a large amount of bias in the difference in the full areas under the curves, the bias correction method reduces the Type I error rate and improves power for the correct decision. We demonstrate the method with an application to a hypothetical oral cancer screening study.

**Conclusion:**

The bias correction method reduces decision errors for some paired screening trials. In order to determine if bias correction is needed for a specific screening trial, we recommend the investigator conduct a simulation study using our software.

## Background

Paired screening trials are common in cancer screening. For instance, one of the designs considered for a planned oral cancer screening study was a paired comparison of the visual and tactile oral exam with the VELscope imaging device [1]. Two recent breast cancer screening studies used a paired design to compare film and digital mammography [2,3].

In paired cancer screening trials, investigators screen all participants with both screening tests. The screening tests may measure a participant’s disease status with error. To ascertain participants’ disease states more definitively, the study investigator tests each participant with a second, more accurate procedure. We refer to the definitive procedure as a reference standard test. In cancer screening, the most accurate reference standard test is biopsy followed by pathological confirmation of disease. Biopsy is painful and invasive and can only be performed on individuals with a visible lesion. Thus, the study investigator determines participants’ disease states as follows. Participants with unremarkable screening test scores on both screening tests enter a follow-up period. Participants with suspicious screening test scores or who show signs and symptoms of disease during follow-up undergo further workup leading to a reference standard test. Participants who do not show signs and symptoms of disease during follow-up are assumed to be disease-free. Follow-up can be thought of as an imperfect reference standard. The reference standard is imperfect because, in truth, some participants may have occult disease.

In the trial by Lewin *et al.*[2], the investigators used a standard analysis to compare the full areas under the receiver operating characteristic curves. The standard analysis includes all participants, even those whose disease status is not verified with the reference standard test. Because some cases may be misclassified, the estimates of diagnostic accuracy may be biased, causing decision errors [4]. If the bias is severe enough, investigators can detect a difference between screening tests when there is none, or conclude incorrectly that the inferior test is superior. Choosing the inferior test can delay diagnosis, increasing morbidity and mortality.

Screening trials are subject to different biases depending on the choice of reference standard and analysis plan [5-8]. Paired screening trial bias [4], the focus of our research, is a special case of differential verification bias. Differential verification bias occurs when 1) a reference standard is used for some participants and an imperfect reference standard is used for the remaining participants, 2) the decision to use the reference standard depends on the screening test results and 3) data from all participants are included in the analysis [7]. Paired screening trial bias occurs in paired studies when the screening tests are subject to differential verification bias and refer different proportions of participants to the reference standard test [4].

We propose a bias-correction method to reduce decision errors in paired cancer screening trials. Under the assumption that the screening test scores follow a bivariate Gaussian distribution, conditional on disease status, we use an iterative, maximum likelihood approach to reduce the bias in the estimates of the mean, variance and correlation. The resulting estimates are then used to reduce bias in the estimates of the diagnostic accuracy of the screening tests.

In the following sections, we describe the bias correction method and evaluate its performance by simulation. In the Methods section, we explain the study design of interest, outline the assumptions and notation, delineate the bias correction algorithm and describe the design of the simulation studies. In the Results section, we report the results of the simulation studies and demonstrate the utility of the method using a hypothetical oral cancer screening study. Finally, in the Discussion section, we discuss the implications of the results and provide recommendations.

## Methods

### Study design

**Figure 1:**
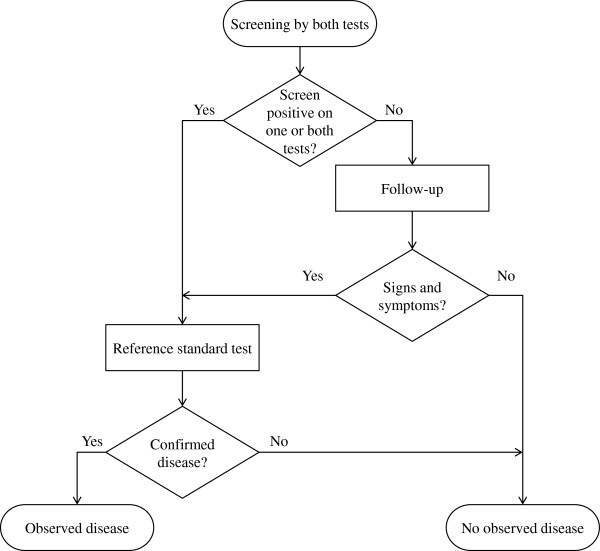
**Flowchart of a paired trial of two continuous screening tests.** The flowchart culminates in the study investigator’s observation of the disease status of the participant.

The study design of interest is a paired study of two continuous cancer screening tests. A flowchart of the study design is shown in Figure 1.

We consider the screening study from two points of view [9]. The first viewpoint is that of the omniscient observer who knows the *true* disease status of each participant. The second viewpoint is that of the study investigator, who can only know the disease status *observed* in the study.

The study investigator determines a participant’s *observed* disease status as follows. Any score that exceeds the threshold of suspicion defined for each screening test triggers the use of a reference standard test. Cases identified due to remarkable screening test scores are referred to as *screen-detected* cases. Participants with unremarkable screening test scores on both screening tests enter a follow-up period. Some participants may show signs and symptoms of disease during the follow-up period, leading to a reference standard test and pathological confirmation of disease. These participants are referred to as *interval* cases. We refer to the collection of screen-detected cases and interval cases as the *observed* cases. Participants with unremarkable screening test scores who do not show signs and symptoms of disease during the follow-up period are assumed to be disease-free, or *observed* non-cases.

**Figure 2:**
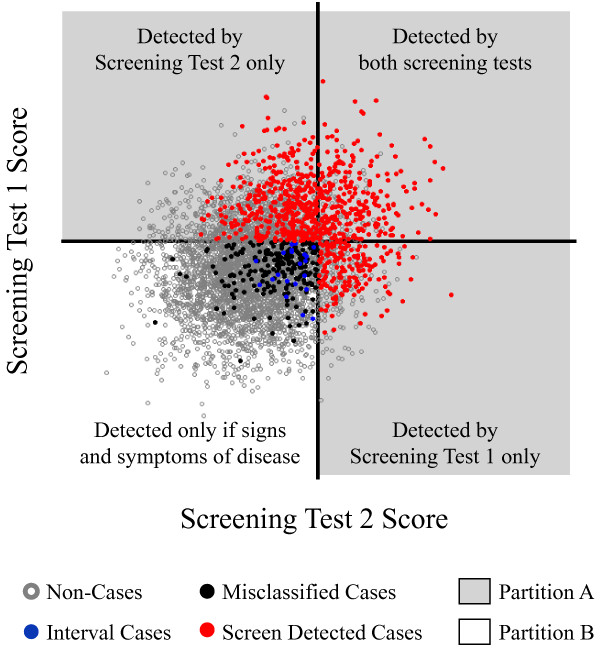
**Hypothetical data for a paired screening trial.** Data in partition *A* (gray) are the set of true cases where at least one screening test score falls above the threshold for that screening test. Data in partition *B* (white) are the set of true cases where the scores on both screening tests fall below their respective thresholds.

Under the assumption that the reference standard test is 100% sensitive and specific, the study design described above will correctly identify all non-cases. However, the design may cause some cases to be misclassified as non-cases. *Misclassified* cases occur when study participants who actually have disease receive unremarkable screening test scores and show no signs or symptoms of disease.We present a graph of a hypothetical dataset of screening test scores (Figure 2) to illustrate how the study investigator observes disease status. The axes represent the thresholds of suspicion for each screening test. We can identify the misclassified cases because we present this graph from an omniscient point of view.

### Standard analysis

In the standard analysis, the study investigator compares the diagnostic accuracy of the two screening tests, measured by the full area under the receiver operating characteristic curve. The goal of the analysis is to choose the screening test with superior diagnostic accuracy.

The receiver operating characteristic curves are calculated using data from all cases and non-cases *observed* in the study. When cases are misclassified, the denominator of the sensitivity decreases while both the numerator and denominator of the specificity increase. As a result, the study investigator overestimates both the sensitivity and specificity of the screening test. The error in sensitivity and specificity causes concomitant errors in the area under the curve. Thus, the *observed* area under the curve can be biased. Paired screening trial bias occurs when the *observed* areas under the curves are differentially biased, causing the difference between the *observed* areas to be either larger or smaller than the true state of nature.

The proposed bias correction method only corrects the estimation of the sensitivity and does not correct specificity. For screening trials using the study design and standard analysis described above, the error in sensitivity may be large [4]. The error in specificity, however, is typically negligible. The large number of non-cases makes the specificity robust to small deviations in the number of *observed* cases. In scenarios with a higher disease prevalence, the error in the uncorrected specificity may affect the performance of the method.

### Assumptions, definitions and notation

We make a series of assumptions. Let *n* be the total number of study participants and *π* the prevalence of disease in the population. Assuming simple random sampling, the number of participants with disease is *M*, and is distributed

(1) M∼Binomial(n,π).

Let *i* index participants, *j* index the screening test and *k* indicate the true presence (*k*=1) or absence (*k*=0) of disease. The pair of screening test scores, *X*_*i*1*k*_ and *X*_*i*2*k*_, are independently and identically distributed bivariate Gaussian random variables with means *μ*_*jk*_, variances $\sigma_{\text{jk}}^{2}$, and correlation *ρ*_*k*_.

Let *a*_*j*_ be the threshold of suspicion for screening test *j*. All scores above the threshold will trigger the use of a reference standard test. For screening test *j*, the *percent ascertainment* is 100 times the number of participants with disease who score above the threshold on screening test *j*, divided by the total number of participants observed to have disease.

Let *I* be the event that a participant shows signs and symptoms of disease and *P*(*I*|*k*=1)=*ψ*. Because participants without the target disease are unlikely to show signs and symptoms of that disease, we assume that *P*(*I*|*k*=0)=0. In practice, however, the clinician must respond to any signs and symptoms with further testing, even if those signs and symptoms may not, in fact, be caused by the target disease. Thus, participants who show signs and symptoms of disease during the follow-up period will still receive the reference standard test and subsequent pathological confirmation.

### Bias correction algorithm

We describe an algorithm to reduce bias in estimates of diagnostic accuracy. The algorithm corrects the maximum likelihood estimates of the parameters of the distribution of case screening test scores. The algorithm then uses a weighting scheme to reduce the variance of the estimates. The corrected maximum likelihood estimates are used to calculate corrected estimates of the diagnostic accuracy of the screening tests.

The algorithm requires four steps.

#### ***Step 1. Partition***

The cases can be stratified into two sets, shown in Figure 2. Let *A* (data in the gray area) be the set of *true* cases with at least one screening test score above its respective threshold. Let *B* (data in the white area) be the set of *true* cases where the scores on both screening tests fall below their respective thresholds. The percentages of participants *observed* to have the disease in sets *A* and *B* differ: all cases in set *A* are observed, but only a fraction of cases are observed in set *B*. The estimation for each set is handled separately in Step 2. Then, in Step 3, the estimates are combined using weighting proportional to the sampling fraction ([10], p. 81, Equation 3.3.1).

#### ***Step 2. Maximum likelihood estimation***

We obtain maximum likelihood estimates of the bivariate Gaussian parameters for the cases. The estimation process follows the iterative method suggested by Nath [11]. The method allows unbiased estimation of bivariate Gaussian parameters from singly truncated convex sample spaces. To obtain singly truncated convex sets, we further partition the sample space into quadrants *Q*_*l*_∈{1,2,3,4}, as shown in Table 1.

**Table 1:** Quadrant definitions

**Quadrant**	**Definition**
*Q*_1_	{*x*_*i*1*k*_≥*a*_1_;*x*_*i*2*k*_≥*a*_2_}
*Q*_2_	{*x*_*i*1*k*_≥*a*_1_;*x*_*i*2*k*_<*a*_2_}
*Q*_3_	{*x*_*i*1*k*_<*a*_1_;*x*_*i*2*k*_≥*a*_2_}
*Q*_4_	{*x*_*i*1*k*_<*a*_1_;*x*_*i*2*k*_<*a*_2_}

The starting values for the iteration are the sample statistics for the *observed* cases in each quadrant. Using the Nath method for each set of starting values results in four sets of quadrant specific maximum likelihood estimates. From the four quadrant specific estimates, we choose the set that maximizes the log likelihood of the full bivariate Gaussian distribution. We refer to that set as the Nath estimates, denoted by ${\hat{\mu }}_{11,N}$, ${\hat{\mu }}_{21,N}$, ${\hat{\sigma }}_{11,N}^{2}$, ${\hat{\sigma }}_{21,N}^{2}$ and ${\hat{\rho }}_{1,N}$.

We require the sample variance as a starting value for the Nath algorithm. Thus, quadrant specific estimates are not calculated for quadrants containing less than two data points.

#### ***Step 3. Weighting***

The Nath estimates are based on only one quadrant of data. We use the process described below to calculate weighted estimates which incorporate data from all quadrants, thereby lowering the variance.

First, the Nath estimates are used as inputs for calculating the sampling fraction for sets *A* and *B*. Define the estimated probability of *A* as

(2) $$\hat{\lambda }=1-\Phi \left(\frac{a_{1}-{\hat{\mu }}_{11,N}}{{\hat{\sigma }}_{11,N}},\frac{a_{2}-{\hat{\mu }}_{21,N}}{{\hat{\sigma }}_{21,N}},{\hat{\rho }}_{1,N}\right)\text{.}$$

Second, the *observed* data are used to calculate the *observed* sample statistics for sets *A* and *B*. The *observed* sample statistics are defined as follows. Let *k*^′^=1 if a participant is observed to have disease and *k*^′^=0 otherwise. For set *s*∈{*A*,*B*}, screening test *j* and *observed* disease status *k*^′^, let ${\bar{X}}_{jk^{\prime },s}$ be the sample mean, $S_{jk^{\prime },s}$ be the sample standard deviation and $r_{k^{\prime },s}$ be the sample correlation between the screening tests.

Finally, the weighted estimates are calculated as a function of the sampling fraction (Equation 2) and the *observed* sample statistics for sets *A* and *B*. We derived expressions for the weighted estimates using the conditional covariance formula ([12], p. 348, Proposition 5.2) and the definition of the weighted mean ([10], p. 77, Equation 3.2.1). Let ${\hat{\mu }}_{j1,W}$, ${\hat{\sigma }}_{j1,W}^{2}$ and ${\hat{\rho }}_{1,W}$ be the weighted estimates of the mean, variance and correlation of the screening test scores for the cases, respectively. We define the estimates as follows:

(3) $$\begin{array}{ll} & {\hat{\mu }}_{11,W}=\hat{\lambda }{\bar{X}}_{11,A}+(1-\hat{\lambda }){\bar{X}}_{11,B}\text{,}\; \end{array}$$

(4) $$\begin{array}{ll} & {\hat{\mu }}_{21,W}=\hat{\lambda }{\bar{X}}_{21,A}+(1-\hat{\lambda }){\bar{X}}_{21,B}\text{,}\; \end{array}$$

(5) $$\begin{array}{ll} & {\hat{\sigma }}_{11,W}^{2}=G_{1}+{ℋ}_{1}-{\hat{\mu }}_{11,W}^{2}\text{,}\; \end{array}$$

(6) $$\begin{array}{ll} & {\hat{\sigma }}_{21,W}^{2}=G_{2}+{ℋ}_{2}-{\hat{\mu }}_{21,W}^{2}\; \end{array}$$

and

(7) $${\hat{\rho }}_{1,W}={\hat{\sigma }}_{\;\;11,W}^{-1}\;{\hat{\sigma }}_{\;\;21,W}^{-1}(P+Q-{\hat{\mu }}_{11,W}{\hat{\mu }}_{21,W})\text{,}$$

where

(8) $$\begin{array}{ll} & G_{j}=\hat{\lambda }\left({\bar{X}}_{j1,A}^{2}+S_{j1,A}^{2}\right)\text{,}\; \end{array}$$

(9) $$\begin{array}{ll} & {ℋ}_{j}=(1-\hat{\lambda })\left({\bar{X}}_{j1,B}^{2}+S_{j1,B}^{2}\right)\text{,}\; \end{array}$$

(10) $$\begin{array}{ll} & P=\hat{\lambda }{\bar{X}}_{11,A}{\bar{X}}_{21,A}+\hat{\lambda }S_{11,A}S_{21,A}r_{1,A}\; \end{array}$$

and

(11) $$Q=(1-\hat{\lambda }){\bar{X}}_{11,B}{\bar{X}}_{21,B}+(1-\hat{\lambda })S_{11,B}S_{21,B}r_{1,B}\text{.}$$

The weighted estimates are the *corrected* estimates used to calculate the *corrected* areas under the receiver operating characteristic curves. If either set *A* or set *B* contain only one observation, we do not conduct the weighting and instead use the Nath estimates as the *corrected* estimates.

Software to implement the method is available at [13].

### Evaluation of bias correction

We compared three methods of analysis: *true*, *observed* and *corrected*. For the *observed* analysis, we used the *observed* sample statistics to calculate estimates of diagnostic accuracy, replicating the standard analysis performed by the study investigator of a cancer screening trial. For the *corrected* analysis, we used the proposed bias correction approach. Finally, both the *observed* and *corrected* analyses were compared to the *true* analysis. In the *true* analysis, we assumed that the study investigator knew the *true* disease status of every participant.

For each analysis, we tested the null hypothesis that there was no difference in the areas under the binormal receiver operating characteristic curves. The areas under the curves were calculated as described in ([14], Equations 12 and 13). We then calculated the variance of the difference in the areas under the curves and conducted a two-sided hypothesis test using the method of Obuchowski and McClish [15].

To assess screening test performance, we compared the Type I error and power of the *observed*, *corrected* and *true* analyses. Because the estimates of diagnostic accuracy can be biased, the study investigator can correctly conclude that there is a difference between the two screening tests but incorrectly choose to implement the screening test with the lower diagnostic accuracy. To quantify this decision error, we divided power into the *correct rejection fraction* and the *wrong rejection fraction*. The correct rejection fraction is the probability that the hypothesis test rejects and the screening test with the larger *observed* area under the curve is the screening test with larger *true* area under the curve. The wrong rejection fraction is the probability that the hypothesis test rejects but the screening test with the larger *observed* area under the curve is the screening test with the smaller *true* area under the curve.

### Design of simulation studies under the Gaussian assumption

Data were simulated per the assumptions listed in the Assumptions, definitions and notation section. We considered two states of nature; one where the null hypothesis holds and one where the alternative hypothesis holds. Under the null, we fixed the *true* areas under the curves to be 0.78. Under the alternative, we fixed the *true* area under the curve to be 0.78 for Test 1 and 0.74 for Test 2 for a difference of 0.04. The sample size was fixed at 50,000. The diagnostic accuracy of the screening tests and the sample size were similar to those in the study by Pisano *et al.*[3]. Except where noted, the correlation between screening test scores for both the cases and non-cases was set to 0.10. Also except where noted, a random sample of 10% of the cases showed signs and symptoms of disease. Recall that showing signs and symptoms of disease only changes the decision to conduct a biopsy if the participant scored negative on both screening tests. The threshold of suspicion for Test 1 was set so that very few cases were referred to the reference standard test. The threshold for Test 2 was set so that nearly all cases were referred to the reference standard test. Different levels of percent ascertainment for each screening test can cause the estimates of diagnostic accuracy to be biased by a different amount [4]. Under the conditions of this simulation study, the differential bias was extreme and, on average, caused the receiver operating characteristic curves to switch orientation relative to the true state of nature.

The simulation studies varied four factors: the disease prevalence, the proportion of cases that exhibited signs and symptoms of disease during follow-up, the correlation between Test 1 and 2 scores and the positions of the thresholds that trigger a reference standard test. The four factors changed the number of *observed* cases and the amount of bias in the estimates of diagnostic accuracy. We set the disease prevalence to 0.01, 0.14 or 0.24, reflecting cancer rates seen in published cancer studies and surveys [2,3,16-18]. The rate of signs and symptoms was varied across a clinically relevant range of 0 to 0.20 [2,17,19]. We examined a range of correlations between 0 and 1. To assess the effects of smaller degrees of differential bias, we set the thresholds of suspicion to result in 15, 50 and 80 percent ascertainment and examined each of the nine possible pairings. Each pair varied the amount and source of the bias (Test 1 or Test 2). Note that percentages are approximate because the case numbers are discrete.

For each combination of parameter values, we simulated paired screening test scores and a binary indicator of *true* disease status. Based on the described study design (Figure 1), we deduced the *observed* disease status. After calculating the *true*, *observed* and *corrected* areas under the curves, decision errors were assessed using the metrics described in the Evaluation of bias correction section. We used 10,000 realizations of the simulated data to ensure that the error in the estimation of probabilities occurred in the second decimal place.

### Design of non-Gaussian simulation studies

Although the bias correction method was developed under an assumption that the data were bivariate Gaussian, screening data may not follow the Gaussian distribution. We conducted a second set of simulation studies to examine the performance of the bias correction method for multinomial and zero-weighted data.

Multinomial and zero-weighted data occur often in imaging studies. Readers may give the image a score of zero to indicate that no disease is seen, resulting in a dataset where multiple values are zero. Reader preferences for a subset of scores can produce multinomial data. To generate the zero-weighted data where the occurrence of zeroes is correlated between the two screening tests, we created two sets of Bernoulli random variables, one for the cases and one for the non-cases, so that the probability that the score on Test 1 is zero is *p*_1*k*_, the probability that the score on Test 2 is zero is *p*_2*k*_ and the probability that both screening test scores are zero is *q*_*k*_. If the Bernoulli random variable was one, we replaced the associated screening test score with a zero. Otherwise, the screening test score remained as it was. We set *p*_*jk*_ equal to a range of values between 0 and 0.90. The marginal probabilities put constraints on the possible values for *q*_*k*_[20]. We set *q*_*k*_ to the median allowed agreement for each pairing of *p*_*jk*_.

To generate multinomial data, we binned the bivariate Gaussian data. Bin sizes ranged from 1/10 to 2 times the standard deviation. Disease prevalence was 0.01, 0.14 and 0.24. All other parameter values were equivalent to those in the Gaussian simulation studies. The performance of the method was evaluated as described in the Evaluation of bias correction section.

## Results

### Overview

**Figure 3:**
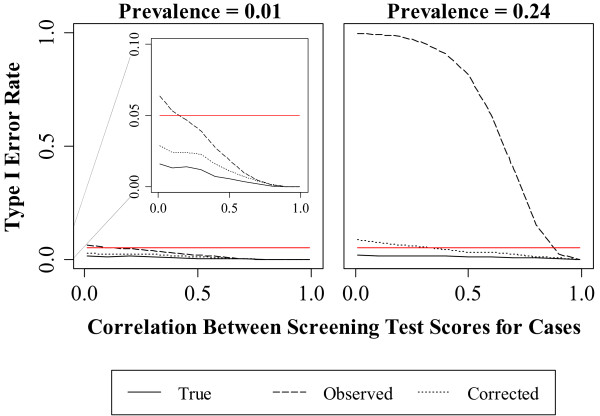
**Effect of case correlation on the Type I error rate.** The nominal Type I error was fixed at 0.05 and is indicated by the red line.

**Figure 4:**
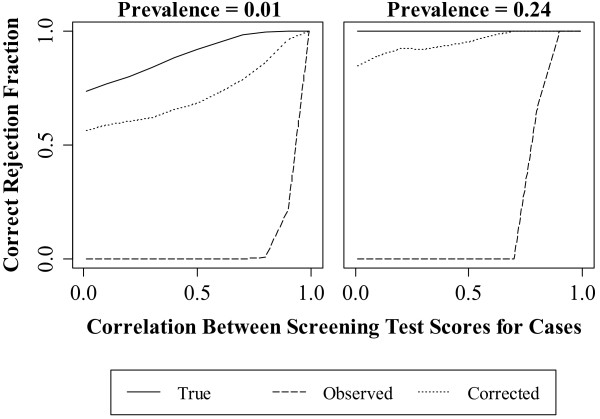
**Effect of case correlation on the correct rejection fraction.** The correct rejection fraction is the proportion of times the hypothesis test rejects when the alternative is true and the choice of the superior screening test is aligned with the true state of nature.

**Figure 5:**
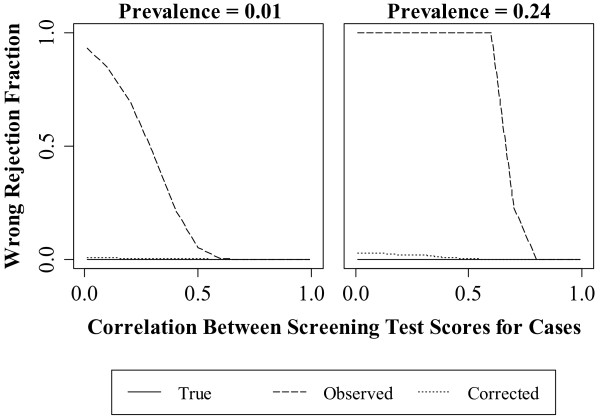
**Effect of case correlation on the wrong rejection fraction.** The wrong rejection fraction is the proportion of times the hypothesis test rejects when the alternative is true and the choice of the superior screening test is opposite the true state of nature.

When compared to the *observed* analysis, the bias correction method reduced decision errors across all experimental conditions where the percent ascertainment differed between the two screening tests (Figures 3, 4, 5 and Table 2, Rows 1-9). However, the Type I error rate for the *corrected* analysis was still above nominal for many experimental conditions (Table 2).

**Table 2:** Effect of percent ascertainment on the Type I error rate

**Paired screening**	**Disease**	**Percent ascertainment**	**True**	**Observed**	**Corrected**
**trial bias**	**prevalence**	**(Test 1/Test 2)**			
	0.01	15/50	0.01	0.89	0.36
	0.01	15/80	0.02	0.95	0.25
	0.01	50/80	0.01	0.23	0.12
	0.14	15/50	0.02	1.00	0.82
Yes	0.14	15/80	0.02	1.00	0.60
	0.14	50/80	0.02	1.00	0.20
	0.24	15/50	0.02	1.00	0.95
	0.24	15/80	0.02	1.00	0.91
	0.24	50/80	0.02	1.00	0.40
	0.01	15/15	0.01	0.02	0.23
	0.01	50/50	0.01	0.02	0.12
	0.01	80/80	0.02	0.02	0.18
	0.14	15/15	0.02	0.02	0.26
No	0.14	50/50	0.02	0.02	0.14
	0.14	80/80	0.02	0.02	0.03
	0.24	15/15	0.02	0.02	0.26
	0.24	50/50	0.02	0.02	0.14
	0.24	80/80	0.02	0.02	0.04

Type I error rates are calculated over 10,000 realizations of the data for the hypothesis test of a difference in the full areas under the curves. The nominal Type I error is fixed at 0.05.

Variations in the disease prevalence, the case correlation and the position of the thresholds of suspicion had the largest effect on the Type I error rate and power of the *corrected* analysis. The difference between the Type I error rate and power of the *corrected* analysis compared to the *true* analysis was only slightly modified by changes in the rate of signs and symptoms (details given in Additional file 1). The non-case correlation is not involved in the bias correction calculations and, as expected, had no effect on the performance of the method.

The bias correction method reduced decision errors when screening test scores had a multinomial distribution with bin sizes up to 1/4 the standard deviation and the disease prevalence was medium or high. However, the Type I error rate was above nominal. The Nath algorithm had high failure rates when more than 1% of screening test scores were zero.

### Effect of disease prevalence and case correlation

As shown in Figure 3, higher disease prevalence resulted in higher Type I error rates for the *true*, *observed* and *corrected* analyses. Type I error declined with increasing case correlation. The Type I error rate of the *corrected* analysis was below nominal at low disease prevalence and decreased from 0.09 to below nominal at high prevalence. The Type I error rate of the *observed* analysis had a high of 0.06 at low prevalence then decreased to below nominal. At high prevalence, the Type I error rate of the *observed* analysis decreased from 0.95 to 0.05.

In Figure 4, higher disease prevalence and case correlation resulted in a higher correct rejection fraction. The correct rejection fraction for the *true* analysis ranged from 0.74 to 1.0 at low prevalence and was 1.0 at high prevalence. The correct rejection fraction for the *corrected* analysis ranged from 0.57 to 1.0 at low prevalence and 0.85 to 1.0 at high prevalence. The correct rejection fraction of the *observed* analysis, however, was 0 except at correlations greater than approximately 0.7 at low prevalence and 0.8 at high prevalence.

In Figure 5, the wrong rejection fraction was at or near 0 for the *corrected* analysis across all experimental conditions. By contrast, the wrong rejection fraction for the *observed* analysis was 1 at low and medium correlation across all disease prevalences. At high correlation, the wrong rejection fraction for all analyses went to zero.

### Effect of percent ascertainment

Table 2 shows the Type I error of the *true*, *observed* and *corrected* analyses for nine pairs of percent ascertainment levels. We do not discuss the power results since the Type I error of the *observed* analysis was so high and power is bounded below by Type I error rate.

In general, when the study had some amount of paired screening trial bias (as indicated by a difference in the percent ascertainment), the Type I error rate of the *observed* analysis was too high (0.23 to 1.0). The Type I error rate of the *corrected* analysis was closer to nominal than that of the *observed* analysis, but was also too high (0.12 to 0.95). For pairings with no paired screening trial bias, the *observed* analysis had lower than nominal Type I error rates while the *corrected* analysis had Type I error rates up to 0.26. When both screening tests had high percent ascertainment (80/80), the Type I error rate of the *corrected* analysis was below nominal.

### Robustness to non-Gaussian data

The results of the non-Gaussian simulation studies are summarized below. A table of the main results is presented in Additional file 2.

At medium and high disease prevalence, the *corrected* analysis had a lower Type I error rate than the *observed* analysis for multinomial bin sizes 1/4 the standard deviation or less. At low disease prevalence, the Type I error rate for the *corrected* analysis was lower than the *observed* analysis for multinomial bin sizes 1/10 the standard deviation or less.

For the range of multinomial bin sizes considered in the study, the Type I error rate of the *true* analysis remained below nominal. The Type I error rate of the *corrected* analysis, however, was above nominal for all disease prevalences and bin sizes greater than 1/10 the standard deviation. The *observed* analysis had an inflated Type I error rate at medium and high disease prevalence. At low disease prevalence, the Type I error rate of the *observed* analysis was below nominal except at a multinomial bin size of 2 times the standard deviation.

For zero-weighted data, the success rate of the Nath algorithm decreased as the percentage of zero scores for the cases increased. At low disease prevalence, when 1% of the cases had zero scores, the Nath algorithm converged for only 33% of the simulated trials. For zero-weights less than 1%, the Type I error rate for all three analyses was above nominal at medium and high disease prevalence. However, the Type I error rate of both the *true* and *corrected* analyses were closer to nominal than that of the *observed* analysis.

### Demonstration

**Figure 6:**
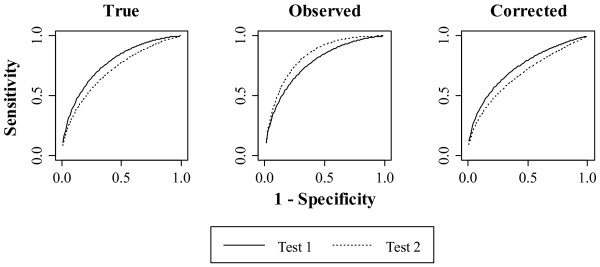
**Receiver operating characteristic curves for a hypothetical oral cancer screening study.** The study is subject to paired screening trial bias. The *true* areas under the curves for Test 1 and Test 2 are 0.77 and 0.71, respectively, for a *true* difference of 0.06. The *observed* difference is -0.06, with the *corrected* difference at 0.06.

Figure 6 shows the receiver operating characteristic curves for a hypothetical oral cancer screening trial similar to that considered by Lingen [1]. One of the designs considered by Lingen was a paired trial comparing two oral cancer screening modalities: 1) examination by a dentist using a visual and tactile oral examination, and referral for biopsy only for frank cancers (Test 1); and 2) examination by a dentist using a visual and tactile oral exam, a second look with the VELscope oral cancer screening device and stringent instructions to biopsy any lesion detected during either examination (Test 2).

We could find no published oral cancer screening trials of paired continuous tests. Instead, we chose parameter values from a breast cancer screening study [3] and an oral cancer screening demonstration study [17]. We fixed the sample size at 50,000 and the rate of visible lesions at 0.1 [17, rate of suspicious oral cancer and precancerous lesions reported in 28 studies between 1971 and 2002 ranges from 0.02 to 0.17, Table 6]. We approximated the disease prevalence as 0.01 based on the number of Americans with cancer of the oral cavity and pharynx [18] and the 2011 population estimate from the U.S. Census Bureau [21]. For the purposes of the illustration, the *true* areas under the curves for Test 1 and Test 2 were fixed at 0.77 and 0.71, respectively.

In the hypothetical oral cancer screening trial, we posit that there would be a large difference in the percent ascertainments for each screening modality. In the first arm, the dentist only recommends biopsy for participants with highly suspicious lesions. Thus, we fixed the percent ascertainment to be very low, only 0.01% of the cases. The oral pathologist recommends biopsy for almost any lesion so we set the percent ascertainment at 97% of the cases. The large difference in percent ascertainment created extreme paired screening trial bias, causing the receiver operating characteristic curves to switch orientation relative to the truth.

When there is an extreme amount of differential bias, the method performs well (Figure 6). The *true* difference in the areas under the curves was 0.06 (p = 0.001) and the *observed* difference was - 0.06 (p = 0.005). The *corrected* analysis realigned the curves with the true state of nature, adjusting the difference back to 0.06 (p = 0.001).

In reality, the study investigator would not know which analysis had results closest to the truth. To validate our choice of analysis, we simulated the hypothetical study using the parameter values specified above. The simulated Type I error rate of the *corrected* analysis was below nominal at 0.03, while the Type I error rate of the *observed* analysis was above nominal at 0.06. The correct rejection fraction of the *corrected* analysis was 0.58, while that of the *observed* analysis was zero. In fact, using the *observed* analysis, the study investigator would wrongly conclude that Test 2 was superior to Test 1 86% of the time. Based on this simulation, we would recommend the study investigator use the results of the *corrected* analysis.

## Discussion

We could find no other methods that attempted to ameliorate paired screening trial bias. Re-weighting, generalized estimating equations, imputation and Bayesian approaches have been proposed to reduce the effect of partial verification bias (*e.g.*, [22-27]). Maximum likelihood methods [28,29] and latent class models [30] have been proposed to estimate diagnostic accuracy in the presence of imperfect reference standard bias. These methods, however, address problems that are quite different than the one we describe. The proposed approach is the only method that attempts to correct the differential misclassification of disease states.

The bias correction algorithm is a maximum likelihood method. Thus, the accuracy of the estimation depends on the number of cases. We do not recommend using the method for studies with a very small number of cases (<500) and interval cases (<5). The performance of the method improves as the disease prevalence and rate of signs and symptoms increase because both factors increase the amount of information (number of cases) used to form the corrected estimates. As the disease prevalence becomes very large, however, the benefits of the increased amount of case information is constrained by the increasing number of non-cases. The non-case parameter estimates are not corrected and add bias to the estimates of diagnostic accuracy.

For the correlation simulation study, the performance of the method depends upon the *observed* difference in the areas under the curves. Under the conditions of the study, the average difference in the *observed* areas under the curves was zero at an approximate correlation of 0.7. At higher correlations, the average *observed* difference was underestimated but agreed with the true state of nature. At lower correlations the bias was more severe: the average *observed* difference was overestimated and opposite the true state of nature. The bias correction method performed best under conditions with a large amount of differential bias. Thus, at low correlations the *corrected* analysis had lower Type I error rates and higher power for the correct decision relative to the *observed* analysis.

The simulation studies demonstrated that the performance of the bias correction method depends on the amount of differential bias in the study. The amount of bias, in turn, depends on fourteen factors: the means, variances and correlations of the test scores, the disease prevalence, the rate of signs and symptoms and the percent ascertainment for each screening test. After analysis of over 170,000 combinations of the parameter values, we were unable to determine a definitive pattern upon which to base recommendations for using a standard versus a bias-corrected approach. We can, however, provide recommendations for two special cases. We suggest that *observed* study results be used if: 1) all participants receive a reference standard test, or 2) the two screening tests under consideration ascertain approximately the same percentage of cases. Both situations are plausible in cancer screening. In a proposed oral cancer screening trial [1], the investigators suggested biopsying all oral lesions, under the argument that oral biopsy was minimally invasive, and diagnosis was difficult without biopsy. The second case occurred in studies comparing digital and film mammography, which have similar recall rates [2,3].

In order to determine if bias correction is indicated for a screening trial that is not a special case, we recommend the investigator conduct a simulation study similar to those described in the manuscript. The simulation software, instruction manual and example code are available at [13]. The software simulates Type I error rate and power for both the standard and bias-corrected analyses in the SAS/IML environment. In addition, the software can perform bias correction for a user-provided dataset should the bias-corrected approach be deemed appropriate.

Under most circumstances, the study investigator should choose the analysis that has the Type I error rate closest to, but not greater than the nominal level, highest correct rejection fraction and lowest wrong rejection fraction. In some contexts, one type of error may be more important than the other. Controlling the Type I error rate is a priority if there is only one study that is going to be performed and patients could be put at harm if the wrong screening test is selected. A small inflation of the Type I error rate might be less important if there is prior knowledge that the null is not true. For example, say a researcher is designing the last study in a series of studies examining complimentary hypotheses. If all previous studies rejected the null hypothesis, then the researcher has prior knowledge that the phenomenon may show an effect. In this situation, the researcher might prioritize the analysis with a slightly higher than nominal Type I error rate in favor of greater discriminatory power under the alternative hypothesis.

Another limitation of the method is the assumption that screening test scores are distributed bivariate Gaussian conditional on disease status. The bivariate Gaussian distribution is the underlying assumption for the binormal receiver operating characteristic curve, a popular form of receiver operating characteristic analysis [31]. We evaluated the robustness of the method to two common deviations from normality: multinomial and zero-weighted data. Based on our simulation studies, we cannot recommend the method for use with datasets where greater than 1% of test scores have zero values. In addition, the method is not recommended for data with multinomial bin sizes greater than 1/4 the standard deviation for medium or high disease prevalence or 1/10 the standard deviation for low prevalence. In future work, the bias correction method could be expanded to handle alternative distributions for the test scores.

This paper provides two contributions to the literature. First, we describe a method to correct for paired screening trial bias, a bias for which there is no other correction technique. Due to the increasing use of continuous biomarkers for cancer detection (see, *e.g.*, [32]), a growing number of screening trials have the potential to be subject to paired screening trial bias. The proposed method will counteract bias in the paired trials and allow investigators to compare screening tests with fewer decision errors. Second, we introduce an important metric for evaluating the performance of bias correction techniques, that of reducing decision errors. We recommend that any new correction method be evaluated with a study of Type I error and power.

## Conclusions

The proposed bias correction method reduces decision errors in the paired comparison of the full areas under the curves of screening tests with Gaussian outcomes. Because the performance of the bias correction method is affected by characteristics of the screening tests and the disease being examined, we recommend conducting a simulation study using our free software before choosing a bias-corrected or standard analysis.

## Competing interests

The authors declare that they have no competing interests.

## Authors’ contributions

BMR conducted the literature review, derived the mathematical results, designed and programmed the simulation studies, interpreted the results and prepared the manuscript. TAA assisted with the literature review and provided expertise on the context of the topic in relation to other work in the field. JTB assisted with the mathematical derivations. SMK provided guidance for the design and programming of the simulation studies. AM improved the software and packaged it for public release. KEM reviewed the intellectual content of the work and gave important editorial suggestions. DHG conceived of the topic and guided the development of the work. All authors read and approved the manuscript.

## Pre-publication history

The pre-publication history for this paper can be accessed here:

http://www.biomedcentral.com/1471-2288/14/37/prepub

## Supplementary Material

Additional file 1 **Effect of the rate of signs and symptoms.** The file contains results for the simulation study examining the effect of varying the rate of signs and symptoms on the Type I error rate and power of the *true*, *observed* and *corrected* analyses. Click here for file

Additional file 2 **Non-Gaussian simulation study.** The file contains the main results for the simulation study examining the robustness of the bias correction method to deviations from the Gaussian assumption. Click here for file
