# Supplementary material for: Reducing decision errors in the paired comparison of the diagnostic accuracy of screening tests with Gaussian outcomes
Source: BMC Med Res Methodol. 2014 Mar 5;14:37. doi: 10.1186/1471-2288-14-37 (PMC4015908; doi:10.1186/1471-2288-14-37)

Additional file 1 - Effect of the rate of signs and symptoms

Overview

We present the results for the simulation study assessing the effect of the rate of signs and symptoms on the performance of the bias correction method. For a description of the simulation study design, refer to the Design of simulation studies under the Gaussian assumption section in the main text.

Effect of the rate of signs and symptoms

In Figure S1, the Type I error rate declined as the rate of signs and symptoms increased. The Type I error rate of the *corrected* analysis was below nominal at low disease prevalence and ranged from 0.06 to 0.14 at high prevalence. The Type I error rate of the *observed* analysis ranged between 0.05 and 0.07 at low prevalence and 0.97 to 1.00 at high prevalence.

In Figure S2, increasing the rate of signs and symptoms had no effect on the correct rejection fraction at low disease prevalence, but improved the correct rejection fraction at high prevalence. The correct rejection fraction for the *true* analysis was 0.77 at low prevalence and 1.00 at high prevalence. The correct rejection fraction for the *corrected* analysis ranged from 0.56 to 0.59 at low prevalence and 0.72 to 0.94 at high prevalence. By contrast, the *observed* analysis had a correct rejection fraction of zero across all prevalences.

In Figure S3, the wrong rejection fraction for the *corrected* analysis is near zero across all rates of signs and symptoms and disease prevalences. For the *observed* analysis, however, the wrong rejection fraction ranged from 0.51 to 1.00.

Under the conditions of the simulation, a study investigator using the *observed* results would either incorrectly decide that the worst screening test was best, or conclude that there was no difference between the two screening tests.

Figure S1 - Effect of the rate of signs and symptoms on Type I error rate

The nominal Type I error was fixed at 0.05 and is indicated by the red line.

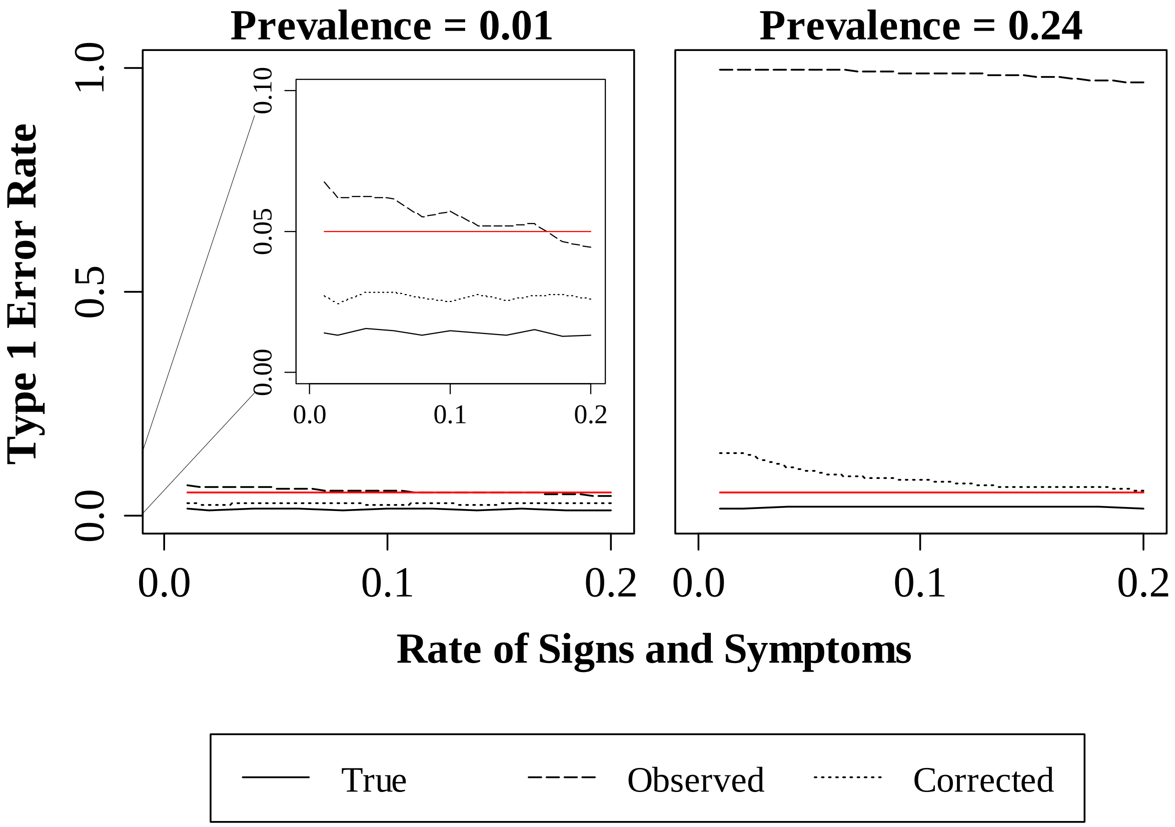

Figure S2 - Effect of the rate of signs and symptoms on the correct rejection fraction

The correct rejection fraction is the proportion of times the hypothesis test rejects when the alternative is true and the choice of the superior screening test is aligned with the true state of nature.

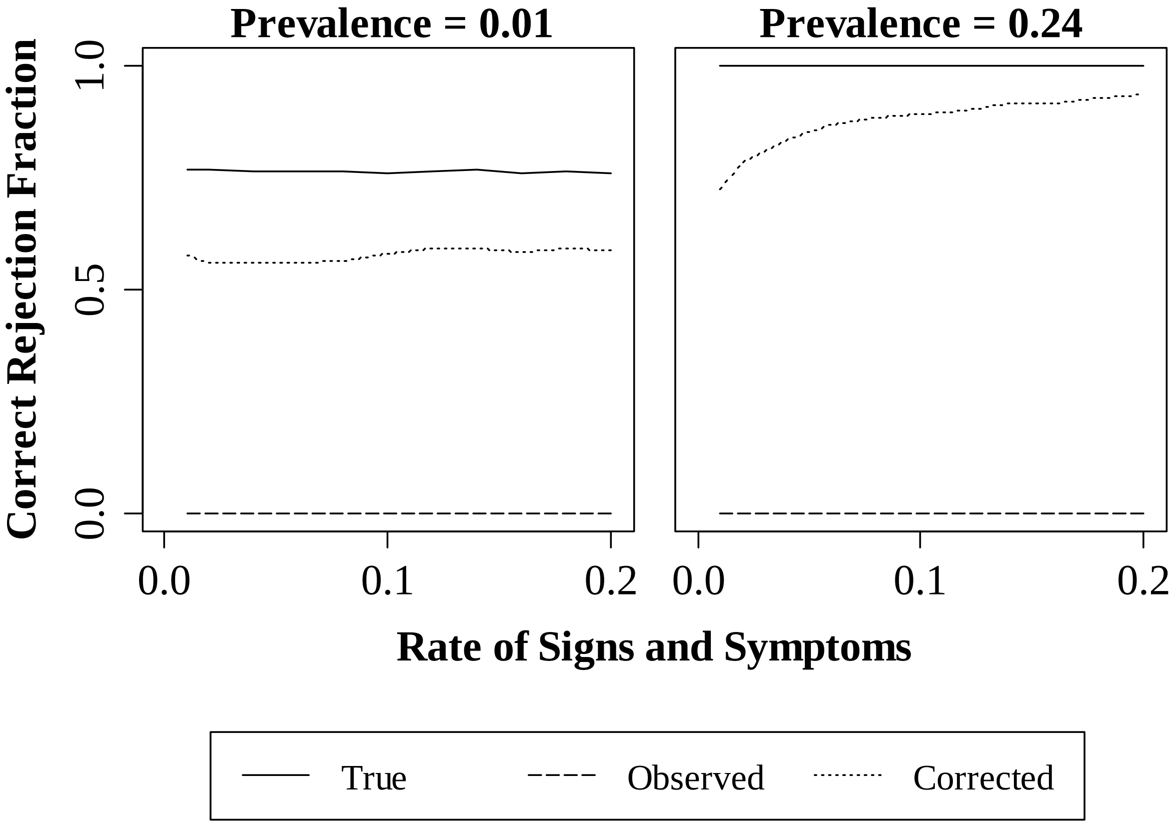

Figure S3 - Effect of the rate of signs and symptoms on the wrong rejection fraction

The wrong rejection fraction is the proportion of times the hypothesis test rejects when the alternative is true and the choice of the superior screening test is opposite the true state of nature.

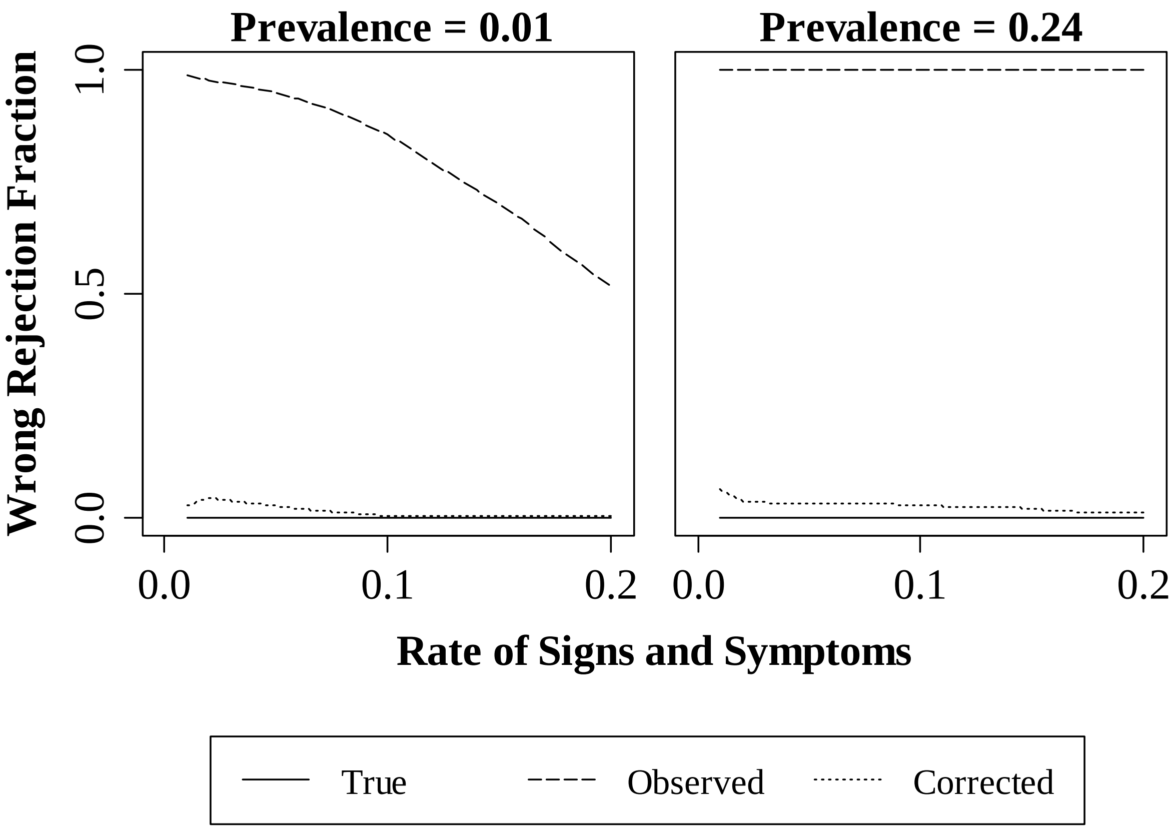

Supplement: Additional file 1 — Effect of the rate of signs and symptoms. The file contains results for the simulation study examining the effect of varying the rate of signs and symptoms on the Type I error rate and power of the true, observed and corrected analyses. [file 1471-2288-14-37-S1.pdf]
