# Supplementary material for: Reducing decision errors in the paired comparison of the diagnostic accuracy of screening tests with Gaussian outcomes
Source: BMC Med Res Methodol. 2014 Mar 5;14:37. doi: 10.1186/1471-2288-14-37 (PMC4015908; doi:10.1186/1471-2288-14-37)
Supplement: Additional file 2 — Non-Gaussian simulation study. The file contains the main results for the simulation study examining the robustness of the bias correction method to deviations from the Gaussian assumption. [file 1471-2288-14-37-S2.pdf]

Additional file 2 - Non-Gaussian simulation study

Overview

We provide a table of important results for the simulation study assessing the robustness of the bias correction method to deviations from the Gaussian assumption. For a description of the simulation study design, refer to the Design of non-Gaussian simulation studies section in the main text.

Tabular Results for the Non-Gaussian Simulation Study

Table S1 displays the effect of increasing the multinomial bin size on the Type I error rate of the *true*, *observed* and *corrected* analyses. Note that bin size is listed as a proportion of the standard deviation of the screening test scores. For example, a bin size of 1/2 indicates that Gaussian screening test scores were rounded to 1/2 the standard deviation to generate the multinomial screening test scores.

For additional information on the results of the non-Gaussian simulation studies, see the Results, Robustness to non-Gaussian data section in the main text.

Table S1 - Effect of multinomial bin size on the Type I error rate of the *true*, *observed*, and *corrected* analyses

Bin Size*	Disease Prevalence	True	Observed	Corrected
1/10	0.01	0.01	0.05	0.03
1/4	0.01	0.01	0.04	0.09
1/2	0.01	0.01	0.03	0.13
1	0.01	0.02	0.02	0.23
2	0.01	0.02	0.09	0.38
1/10	0.14	0.02	0.79	0.05
1/4	0.14	0.02	0.63	0.20
1/2	0.14	0.02	0.38	0.76
1	0.14	0.02	0.11	0.98
2	0.14	0.02	0.94	1.00
1/10	0.24	0.02	0.97	0.05
1/4	0.24	0.02	0.91	0.20
1/2	0.24	0.02	0.68	0.83
1	0.24	0.02	0.23	1.00
2	0.24	0.03	1.00	1.00

*Relative to the standard deviation of the screening test scores.
